# Advancements in catheter tip shape and technology: The way forward

**DOI:** 10.1002/joa3.12909

**Published:** 2023-08-07

**Authors:** Jose Antonio L. Bautista, Chin‐Yu Lin

**Affiliations:** ^1^ Heart Rhythm Center, Department of Cardiology Taipei Veterans General Hospital Taipei City Taiwan; ^2^ Section of Clinical Cardiac Electrophysiology Heart Institute, St. Luke's Medical Center – Global City Taguig City Philippines; ^3^ Medical department National Yang‐Ming Chiao‐Tung University Taipei Taiwan

The advancing technologies in catheter ablation of various cardiac arrhythmias have been progressing for several years. From the inception of irrigated ablation catheters to improve the depth and delivery of lesions to the introduction of contact force (CF) sensing technology in various ablation catheters to improve catheter‐tissue contact and prevent complications, ablation technologies have evolved significantly, and various studies have been undertaken and taken catheter ablation technologies and strategies to greater heights. However, improving ablation technologies and innovations are similar to opening a Pandora's Box—meaning, for every advancement of ablation technology, either a new or well‐known inadvertent complication related to that emerging catheter technology is unearthed. This prompts researchers to push the bounds of science further, prompting ever‐changing innovations in the advancements in catheter tip shape and technology.

In a published article by Matsumoto and colleagues^1^ on a novel CF‐sensing catheter with a mesh‐shaped irrigation tip, the authors performed an extensive analysis of this innovative catheter, also known as the TactiFlex™ SE (Abbott). This novel catheter was tested on a fresh swine left ventricle slice, and various ablation energies and CF steps were applied using the novel catheter on the experimental model. The researchers then systematically assessed the characteristics of the lesions being formed by this novel catheter and compared this with a previously known mesh‐shaped ablation catheter that did not contain any CF‐sensing technology (FlexAbility™ SE, Abbott). From the results of the study, the researchers were able to discover the following: First, the lesion formation between these two mesh‐shaped ablation catheters was almost similar for the various CF and wattage settings under a 60‐s ablation. Also, there was no significant difference in the drop in impedance between the two catheters. Another was that steam pops did not exhibit a significant difference between the two ablation catheters, and that a higher CF was related to a higher incidence of steam pops. In conclusion, this novel mesh‐shaped ablation catheter was similar to the FlexAbility™ SE ablation catheter in terms of lesion formation and steam pop incidence.

From the results of this study, the use of the TactiFlex™ SE ablation catheter can help improve ablation delivery with a focus on lessening complications, although impedance drops and incidences of steam pops were not different with FlexAbility™ SE ablation catheter that was used for comparison. It should be taken into a fact that this novel iteration by Abbott is a so‐called “marriage” between the FlexAbility™ SE and a CF‐sensing ablation catheter by the same company (TactiCath™ SE, Abbott) – combining two innovative technologies into one ablation catheter. Combining one technology (mesh‐shaped catheter tip) with another (CF‐sensing technology) definitely provides the advantage of detecting the force of contact between the ablation catheter and myocardial tissue. However, looking at the results of this study, shows that findings in terms of complications like steam pops were not different from that of its predecessor. It must be taken into consideration that CF plays a vital role in preventing steam pops. That is why, although findings were not as different, the use of this novel catheter can still be advantageous because of an important trump card in its deck—the CF‐sensing technology. Several papers have already discussed the advantages of CF‐sensing technology on ablation safety and outcome. This is why advancements in catheter technology are essential in the improvement of ablation delivery strategies with a focus on patient safety and procedural success.

As what was mentioned, advancements in catheter ablation technologies have taken the ablation of various tachyarrhythmias to greater heights. The next step to determine the efficacy and safety of this novel catheter technology is to use this catheter in human subjects. Determining its efficacy and safety in real‐world use will definitely bring the developing study of this ablation technology further toward its zenith—in other words, closer toward the peak of its success. Therefore, further studies on this and other novel catheter ablation technologies are the way forward.
